# Effects of Different Inspiratory Muscle Training Protocols on Exercise Capacity, Respiratory Muscle Strength, and Health-Related Quality of Life in Patients with Hypertension

**DOI:** 10.1155/2024/4136457

**Published:** 2024-02-03

**Authors:** İrem Hüzmeli, Nihan Katayıfçı, Fatih Yalçın, Esra Doğru Hüzmeli

**Affiliations:** ^1^Hatay Mustafa Kemal University, Faculty of Health Sciences, Department of Physiotherapy and Rehabilitation, Hatay, Türkiye; ^2^Hatay Mustafa Kemal University, Faculty of Medicine, Department of Cardiology, Hatay, Türkiye

## Abstract

**Aim:**

This study aimed to explore how varying inspiratory muscle training workloads affect exercise capacity, health-related quality of life (HrQoL), depression, peripheral and respiratory muscle strength, pulmonary function, dyspnea, fatigue, and physical activity levels in hypertension (HT) patients.

**Methods:**

A randomized, controlled three-arm study. Forty-five patients (58.37 ± 8.53 y, 7F/38M) with HT received IMT (7 days/8 weeks) by POWERbreathe® Classic LR device and were randomized to control group (CG, 10% maximal inspiratory pressure (MIP), *n*: 15), low-load group (LLG, 30% MIP), and high-load group (HLG, %50 MIP). Exercise capacity, HrQoL, depression, peripheral and respiratory muscle strength, pulmonary function, fatigue, physical activity level, dyspnea, and sleep quality were evaluated before and after the training.

**Results:**

Exercise capacity, physical functioning, peripheral muscle strength, and resting dyspnea were statistically significantly improved in HLG and LLG after the training compared to CG (*p* < 0.05). Similar improvements in perception of depression, fatigue, and sleep quality were seen within and between the groups (*p* > 0.05). Statistically significant differences were found within all the groups in terms of MIP and PEF values of respiratory functions (*p* < 0.05). The superior improvement in the physical activity level was found in the HLG (*p* < 0.05). *Discussion*. High-load IMT was particularly effective in increasing physical activity level, peripheral muscle strength, exercise capacity, and improved HrQoL. Low-load IMT was effective in reducing dyspnea and improving respiratory function. Device-guided breathing exercises decreased blood pressure, improved sleep quality, and strengthened respiratory muscles. IMT, an efficient method, is suggested for inclusion in rehabilitation programs due to its capacity to increase physical activity, exercise capacity, and peripheral muscle strength, enhance HrQoL and respiratory function, and alleviate dyspnea. Also, the efficacy of IMT should be investigated with different training protocols such as endurance IMT or functional IMT in HT patients.

## 1. Introduction

Hypertension, a prevalent chronic condition, affects over a billion individuals globally, elevating the risks of cardiovascular, cerebrovascular, and renal diseases. Its prevalence rises with age, resulting in substantial healthcare expenses [1]. Increases in blood pressure above 115 mmHg (systolic) and 75 mmHg (diastolic) worsen the prognosis [2, 3]. A vigorous public awareness campaign is needed to alleviate the burden of HT and its complications. Besides medication, it is advised to consider the following: (1) decrease sodium intake, (2) prevent obesity and weight gain, (3) enhance physical activity, (4) raise disease awareness, and (5) better manage blood pressure while educating about HT and its multisystem complications [4]. It is therefore not surprising that more attention is being paid to the role of nonpharmacological interventions. Lifestyle modifications proven to lower blood pressure include quitting smoking, reducing salt intake, weight loss, increased physical activity, self-monitoring dietary changes, and adopting strategies to enhance adherence to a healthy lifestyle. [4]. In addition, respiratory interventions have also been suggested to reduce BP. Inspiratory muscle training (IMT) is a respiratory training modality in which patients breathe against a load calculated from their maximum static inspiratory pressure. Among hypertensive patients who received inspiratory muscle training (IMT) at 30% of maximum inspiratory pressure (MIP) over 8 weeks, there was an observed decrease in systolic and diastolic blood pressure levels during the daytime (−7.9 and −5.5 mmHg, respectively). Additionally, this intervention led to reduced sympathetic modulation and increased parasympathetic modulation [5]. Ferreira et al. showed that similar to aerobic training, IMT reduced sympathetic activity and improved endothelial function in controlled hypertensive patients after 12 weeks of training [6]. Inspiratory muscle training has been shown to attenuate metaboreflex in healthy subjects [7] and people with chronic heart failure [8]. In healthy subjects, IMT increased exercise time on a bicycle ergometer by 14% [9]; a similar intervention has also been shown to alleviate quadriceps fatigue during exercise [10]. Nagy et al. [11] found that exercise capacity was associated with depression, fatigue, and physical function. Research on the efficacy of breathing exercises for hypertensive patients highlights their ability to lessen stress, anxiety, and depressive symptoms, enhance wellbeing and sleep quality, and offer general beneficial effects [12]. However, there are few studies on the effectiveness of inspiratory muscle training in HT patients. In addition, the effective inspiratory workload to improve exercise capacity, HrQoL, and peripheral muscle strength of HT patients is unclear. In this study, we aimed to explore how varying inspiratory muscle training workloads affect exercise capacity, health-related quality of life (HrQoL), depression, peripheral and respiratory muscle strength, pulmonary function, dyspnea, fatigue, and physical activity levels in hypertension (HT) patients.

## 2. Materials and Methods

HT patients who were followed and treated in the Cardiology Department of Hatay Mustafa Kemal University Research and Application Hospital were included in the training program. We calculated that the study should include a total of 45 HT patients by taking into account the maximum inspiratory pressure (MIP) value of a similar study in the literature [5], with 80% power and 5% type 1 error and dropout from the study.

The inclusion criteria are as follows:

1. Being in an age range of 18–79 years
2. Having a hypertension diagnosis
3. Being in a stable clinical status for the last month

The exclusion criteria are as follows:

1. Having neurological, orthopedic, and chronic respiratory diseases and active malignancy
2. Having unstable heart disease, NYHA (New York Heart Association) IV heart failure, or uncontrolled hypertension
3. Having acute infection
4. History of organ or bone marrow transplantation
5. Having a hemoglobin level <9 g/dL
6. Being pregnant or lactating

The study with protocol number 22/04/2021-17 was approved by the University's Clinical Research Ethics Committee. The Declaration of Helsinki was followed in the conduct of this investigation. Before each participant took part, they all signed informed consent papers.

### 2.1. Study Protocol

The current study has three arms and is prospective, randomized, and controlled. Computer-based block randomization was used to allocate patients at random to the control group (CG, *n*: 15), low-load group (LLG, *n*: 15), or high-load group (HLG, *n*: 15). Individuals diagnosed with HT were evaluated at different times and included in the training program. CG performed IMT 10% of MIP, LLG 30%, and HLG 50% of MIP during seven days/week, with different loads of the respiratory muscle device for seven days/week for eight weeks. For eight weeks, groups received training for 30 minutes a day and seven days a week. The diaphragm and rib cage muscles were strengthened during training by utilizing a pressure threshold-loading device (POWERbreathe VR Classic Low Resistance, IMT Technologies Ltd., Birmingham, UK), which is used to inhale against the same pressure load during each inhalation. Prior to training, patients were instructed to develop adequate breathing skills during a one-week familiarization period. Each group's participants underwent six sessions at home and one session under close supervision in the cardiology clinic (seven days/week). Throughout sessions, patients' vital signs were observed. Each patient received individual care during the program, with a focus on monitoring. Daily record charts were required and reviewed to emphasize training control. Regular calibration, follow-up, and device control occurred at specified intervals. Patients were instructed not to alter their pressure loads.

### 2.2. Measured Parameters

All groups underwent pre- and posttest evaluations at baseline and at the end of the 8th week.

Demographic, physical, and physiologic characteristics were recorded by asking the patients and from the patient file. Exercise capacity was evaluated with the 6-minute walk test (6MWT). ATS (American Thoracic Society) criteria were taken into consideration and the subjects rested for at least half an hour before starting the test. Participants walked briskly at their own walking pace for 6 minutes on a 30-meter straight corridor. The distance reached at the end of the test was recorded in meters [13]. HrQoL was evaluated with SF-36V2. SF-36V2 has eight subparameters: “pain, physical functionality, general health perception, emotional role limitations, physical role limitations, social functionality, energy/fatigue, and mental health” [14]. The Hospital Anxiety and Depression Scale (HADS) was used to assess the depression. The cut-off score of depression and anxiety subscales was 8 [15]. Peripheral muscle strength and hand grip strength were evaluated with a portable dynamometer, and the values obtained were recorded in Newton (N)/kgf/Pascal. Peripheral muscle strength was measured seated, employing a hand-held dynamometer (J-Tech Power Track Commander, Baltimore, MD, USA). This device, known for its portability and cost-effectiveness, serves as an alternative to isokinetic machines and offers greater sensitivity in detecting changes in muscle strength compared to manual muscle tests [16–18]. Respiratory muscle strength “maximum inspiratory pressure (MIP) and maximum expiratory pressure (MEP)” was calculated with a portable, electronic mouth pressure measuring device. The best of the measurements was recorded for analysis [19]. The pulmonary function test was measured with a portable spirometer (Spirobank II® Moggiolino, Rome, Italy). The best of 3 technically acceptable maneuvers with 95% agreement was selected and recorded [20]. The Fatigue Severity Scale used for fatigue assessment was scored between 9 and 63. A score of 36 or higher indicates severe fatigue [21]. Physical activity level was measured with the International Physical Activity Questionnaire (IPAQ). The international validity and reliability studies of this questionnaire were performed by Craig et al. [22]. In this questionnaire, which provides information about the time spent in sitting, walking, moderately vigorous activities, and vigorous activities, the criterion for evaluating all activities is that each activity is performed for at least 10 minutes at a time. A score is obtained as “MET-minutes/week” by multiplying minutes, days, and MET values (multiples of resting oxygen consumption) [22]. Dyspnea was recorded using the Modified Medical Research Council (mMRC) Dyspnea and Borg Dyspnea Scales. Patients rate their level of dyspnea at rest and/or when exercising on a subjective measure called the Modified Borg Scale (MBS), which ranges from 0 to 10. The lowest score of 0 indicates “none” and the highest score of 10 indicates “very severe” dyspnea. The mMRC is a scale used to assess breathlessness during activities of daily living. Patients select the statement that best describes their perceived shortness of breath from among 5 statements about shortness of breath. Scoring is performed on a 0–4 scale [23, 24]. The Pittsburgh Sleep Quality Index (PSQI) was developed by Buysse et al. [25]. The PSQI, which evaluates sleep quality during the last month, includes a total of 24 questions. The total score has a value between 0 and 21. A total score higher than 5 indicates poor sleep quality [25].

### 2.3. Statistical Analysis

All statistical analyses were performed using SPSS version 26. The Shapiro–Wilk test was used to assess the normality of the data. According to the data normality, continuous variables are expressed as mean and standard deviation (SD)/median (IQR), and categorical variables are expressed as number and percentage (%). One-way ANOVA and the Kruskal–Wallis test were used to compare the three groups' baseline characteristics. By controlling for any changes in baseline variables, we applied the ANCOVA (analysis of covariance) test to determine differences in exercise capacity, HrQoL, peripheral and respiratory muscle strength, anxiety and depression, fatigue, sleep quality, dyspnea, and resting blood pressure between groups. For the purpose of using ANCOVA, all presumptions (normally distributed, homogeneous, homogeneity of regression slope, random independent samples, and linearity) have been confirmed. Each outcome measure's baseline values were used as covariates. Calculations were made for post hoc comparisons using the Bonferroni test (syntax). A *p* value <0.05 was considered statistically significant. The remaining study groups' pairwise comparisons and between-group effects were the main topics of the secondary analysis. Intention-to-treat analysis was performed for missing data. By assessing individuals based on their originally assigned groups, the intention-to-treat (ITT) approach allows for impartial conclusions about intervention effectiveness. Its adherence to randomization safeguards its advantages, setting it apart from alternative analysis methods [26]. The ITT analysis facilitates comparing individuals in both the experimental and control groups based on their random assignment to these groups [27].

The effect size (ES) was calculated with Cohen *d* using the results of 6MWT distance and MIP. The magnitude of effect sizes was classified as small (0.2 ≤ d < 0.5), medium (0.5 ≤ d < 0.8), and large (0.8 ≤ d < 1.2) [28, 29].

## 3. Results

**Figure 1:**
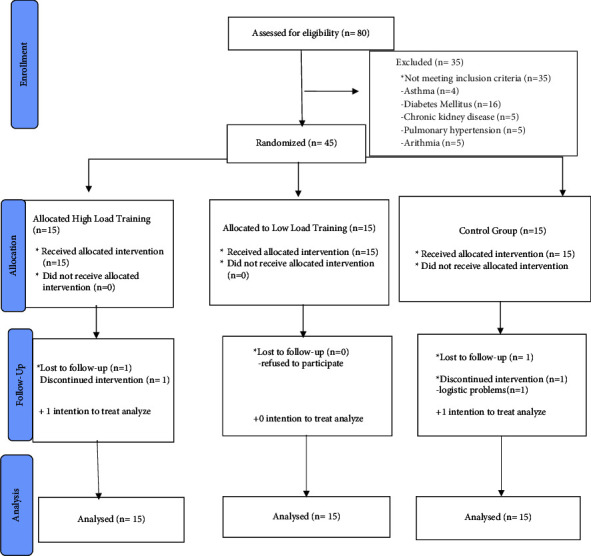
CONSORT flow diagram of the study.

Eighty patients in total had their eligibility for the inclusion criterion checked. Three groups of 45 patients each were randomly assigned. 35 patients in total were excluded, and 43 patients successfully finished the course.  Figure 1 displays the withdrawal's justifications. There were no adverse effects. The patients' prescriptions were not modified during the training.

Sociodemographic and clinical characteristics of individuals diagnosed with HT are shown in Table 1. There was no statistically significant difference between the groups in terms of age (*p* > 0.05). The rate of males was higher than females within the groups and the proportion of males and females was similar between the groups. Physical activity status was similar in all three groups and more than half did not engage in physical activity.

The results of the effect of IMT on respiratory parameters in individuals with HT are shown in Table 2. The results of MIP before and after training were statistically increased within the groups (*p* < 0.05). The effect of IMT between the groups was statistically similar for MIP and MEP (*p* > 0.05). MEP values were statistically increased after the training within LLG and HLG (*p* < 0.05). ∆MIP difference was 12.81 cmH_2_O (*p*=0.290) between the control and LLG, 18.63 cmH_2_O (*p*=0.056) between the control group and HLG, and 5.819 cmH_2_O (*p*=0.999) between HLG and LLG. Medium effects were found in MIP after the training between the groups (ES = 0.570).

The results of FEV_1_ (lt), FEV_1_ (%), FEV_1_/FVC (%), FVC (%), FEF_25–75_ (lt), and FEF_25–75_ (%) from respiratory functions were similar within and between the groups in terms of pre- and post-training values. FVC (lt) was statistically different within LLG and HLG in terms of pre- and posttest values. PEF (lt) pre- and posttest results were statistically different within CG and LLG (*p* < 0.05). A statistically significant improvement was observed across all groups in the PEF (%) results (*p* < 0.05).

Table 3 displays how IMT affects HT patients' peripheral muscular strength, exercise capacity, HrQoL, depression, anxiety, sleep quality, and level of physical activity. The increase in pre- and post-training values in LLG and HLG was statistically significantly different for quadriceps muscle strength, hand grip strength, and 6MWT distance. The effect of training was statistically significant with an increase in the right-hand grip strength and 6MWT distance (*p*<0.05). ∆ 6MWT difference was 21.68 m between CG and LLG (*p*=0.018) and 23.17 m (*p*=0.012) between CG and HLG. There was no statistically significant difference between LLG and HLG in ∆ 6MWT (∆ = 1.48 m, *p*=0.865), but a medium effect (ES = 0.716) was found between the groups in ∆ 6MWT after the training. Physical function and pain pretest and posttest results of SF-36 subparameters were significantly different in LLG and HLG. Social functioning and emotional role difficulty scores of SF-36 were significantly different within HLG compared to the pretraining. The vitality and emotional well-being scores of SF-36 were statistically significantly improved only within LLG. The effect of training was statistically significant in right-hand grip strength, 6MWT distance, and physical function parameter of SF-36 (*p*<0.05). Depression, anxiety, fatigue, and sleep quality results were statistically similar within and between groups (*p*>0.05). Fatigue levels were similar between the groups after the training. The perception of fatigue was similar within CG and HLG compared to the pretraining, whereas the decrease in the perception of fatigue was statistically different within LLG. The increase in physical activity was significant between the groups and the highest increase was found in the high-intensity training group. IMT decreased resting blood pressure, resting dyspnea perception, and improved sleep quality.

## 4. Discussion

This first comprehensive study was aimed to investigate the effects of different IMTs applied for 8 weeks on exercise capacity, HrQoL, depression, peripheral and respiratory muscle strength, respiratory function, fatigue, and physical activity level in HT patients. The most important results of this study are as follows: (1) low-load and high-load IMT increased exercise capacity and improved physical function parameter of HrQoL, (2) although different load protocols of IMT caused a decrease in depression perception, the effects on depression as the no-load were similar, (3) quadriceps muscle strength and hand grip strength improved after training in high-intensity and low-intensity groups, (4) MIP increased in all training loads and the increase was highest in the high load. MEP increased more in the low load and high load, (5) FVC increased mostly in the high load, and a positive change was observed in PEF values with all loads, while similar changes were observed in FEV_1_ and FEV_1_/FVC values, (6) the increase in physical activity was the highest with high-intensity load, and (7) IMT decreased resting blood pressure, resting dyspnea, and fatigue perception and improved sleep quality.

### 4.1. Exercise Capacity

Both healthy and active people can experience a variety of limitations due to weak respiratory muscles. Similar to this, research has demonstrated that the strength of the inspiratory muscle plays a significant role in the pathophysiology of exercise limitation in a variety of clinical disorders [30, 31]. More recent research has confirmed the impact of inspiratory muscles on functional capacity [30] by demonstrating a substantial correlation between MIP and peak VO2 in individuals with acute myocardial infarction and heart failure [32]. HT is one of the increasingly common risk factors globally. Inspiratory muscle weakness has been found in HT patients and this also affects exercise capacity [33]. Studies on the exercise capacity effectiveness of respiratory exercises, one of the alternative treatments of hypertension, have been increasing recently. Ublosakka-Jones et al. [34] found that respiratory exercise training given at low load (MIP 25%) for 8 weeks was effective on arm endurance capacity. In another study examining the effects of respiratory muscle endurance training on performance capacity in normotensive elderly patients, respiratory muscle endurance training applied for 5 weeks caused an increase in performance [35]. Inspiratory muscle training applied at 30% of MIP in pulmonary hypertension patients was not different from the training given at the lowest load of the device in terms of 6MWT results [36]. A similar therapeutic effect was declared on inspiratory muscle strength in COPD patients who performed threshold IMT and resistive IMT at an intensity of 60% of MIP, but resistive IMT was more effective than threshold IMT in improving exercise capacity, inspiratory muscle strength, HrQoL, and degree of dyspnea [37]. Another study concluded that inspiratory muscle strength training at 50% of maximal inspiratory pressure and endurance training at 30% of MIP 7 days/8 weeks were similarly effective on exercise capacity, peripheral muscle strength, respiratory muscle strength, and physical activity level in pacemaker patients with HT [38]. Contrary to the literature, in our study, a significant increase was found in the low-load intensity (MIP 30%) and high-load intensity (MIP 50%) groups compared to the control group (at the lowest load of the device). ∆ 6MWT distance between CG and LLG (21.68 m) and HLG (23.17 m) was statistically significant after the training. The difference between the LLG and HLG was 1.48. The effect of training was statistically significant and medium effective (ES = 0.716) in exercise capacity. It seems that a workload applied to the inspiratory muscles for 8 weeks, whether low or high intensity, may increase exercise capacity. Our study was only related to strength training. Studies examining the effects of high-intensity intermittent inspiratory training/inspiratory endurance training in HT patients are recommended.

### 4.2. Health-Related Quality of Life

HrQoL of individuals with hypertension is slightly worse than normotensive individuals [39]. Aerobic exercises have positive effects on HrQoL. It was found that pulmonary rehabilitation applied for 10 weeks improved HrQoL and increased exercise capacity in individuals with pulmonary hypertension [40]. In another study, IMT increased exercise capacity and MIP in pulmonary hypertension, but there was insufficient information on its effect on HrQoL and fatigue. In a previous study examining the effect of breathing exercises combined with relaxation exercises on cardiovascular risk factors, it was found that relaxation training combined with breathing exercises improved HrQoL [41]. There are no studies in the literature examining the effect of IMT on HrQoL in hypertensive patients. In our study, it was observed that IMT caused an improvement in HrQoL, especially on the physical function parameter. The observed significant enhancement in the PF parameter of the quality of life questionnaire within the training groups possibly contributed to a reduction in perceived exertional dyspnea during daily physical activities, indicating a treatment effect. A previous study has consistently demonstrated that IMT diminishes exertional dyspnea in both healthy and patient populations [42]. This could be attributed to enhancements in peripheral and respiratory muscle strengths, thereby augmenting the overall performance of these muscle groups, a notion supported by studies [42, 43]. These studies illustrate that low-intensity IMT bolsters respiratory muscle strength and functionality across diverse patient groups, encompassing heart failure [44] hemodialysis patients [45], individuals with metabolic syndrome [43], and those with atrial fibrillation [46]. Furthermore, an increase in physical activity tolerance post-IMT might offer an additional rationale for the observed improvement in HrQoL in the present study.

### 4.3. Depression

Respiratory exercises impact physiological mechanisms connected to respiration, emotion, and cognition via the autonomic nervous system. These exercises reduce sympathetic nervous system activity while enhancing parasympathetic nervous system activity, which is linked to cardiac vagal tone. Consequently, these exercises influence emotions, emotional regulation, psychological adaptation, reactivity, expression, and empathic responses. By utilizing this mechanism, conditions such as depression can potentially benefit from breathing exercises [47, 48]. Breathing control has been shown to be beneficial in lowering blood pressure in HT patients [49]. It is not known whether qigong reduces blood pressure by this mechanism, but qigong has been shown to improve respiratory efficiency for oxygen uptake and carbon dioxide production [50]. According to another study in adults aged eighteen years and older, diaphragmatic breathing can reduce stress as measured by physiological biomarkers as well as psychological self-report instruments. Given the benefits of diaphragmatic breathing on stress reduction, further research is needed to continue to build the evidence base for this self-administered, low-cost, nonpharmacological intervention. In our study, device-assisted respiratory exercises performed with or without a certain workload yielded similar results in the perception of depression in HT patients. However, the highest decrease in depression perception was in the LLG and HLG. It is recommended to further investigate the effects of respiratory muscle endurance training on depression in HT patients.

### 4.4. Peripheral and Respiratory Strength

According to a study examining the respiratory-sympathetic connection in relation to hypertension, the respiratory and circulatory systems are both involved in the delivery of oxygen and removal of carbon dioxide from tissues in the body, and any alteration in this connection is expected to lead to cardiovascular consequences. Indeed, studies on this connection have suggested that an increase in respiratory modulation of sympathetic vasoconstrictor drive may contribute to the development of hypertension and that this increased respiratory-sympathetic connection may be responsible for the occurrence of HT in humans [51, 52]. As part of this connection, we found that IMT increased both respiratory muscle strength and quadriceps muscle strength. The clinical significance of these findings may increase the effectiveness of respiratory muscle training in HT. IMT suggests that the reduction in respiratory rate reduces blood pressure through positive modulation of cardiovascular reflexes. This training applies an external resistance to the respiratory muscles and has shown beneficial training effects in patients with cardiovascular disease, especially in patients with chronic heart failure [38]. However, there are very few studies on the effectiveness of IMT on hypertension. According to Ferreira et al. [5], who investigated the effect of 8 weeks of inspiratory muscle training on blood pressure and respiratory muscle strength in HT, there was a significant increase in inspiratory muscle strength in the IMT group (82.7 ± 28.8 vs. 121.5 ± 21.8 cmH_2_O) and this increase was not detected in the placebo-IMT group (93.3 ± 25.3 vs. 106.1 ± 25.3 cmH_2_O). Similar to this study, the difference in ∆MIP between the control and LLG was 12.81 cmH_2_O, between the CG and HLG (MIP 50%) was 18.63 cmH_2_O, and between the HLG and LLG was 5.819 cmH_2_O. The most effective workload that increased respiratory muscle strength was found in the HLG. To increase respiratory muscle strength in HT patients, a respiratory muscle training that includes a workload at 50% of the MIP is recommended.

### 4.5. Respiratory Function

Studies showing the effect of hypertension on the lungs are limited and contradictory. Some investigators show a decrease in pulmonary function parameters in hypertensive patients [53]. The alleged pathophysiology was the occurrence of edema of the lung secondary to left ventricular failure with high sustained blood pressure and reduced elasticity of the pulmonary parenchyma [53, 54]. In contrast, some other researchers have concluded that hypertension has no effect on the pulmonary function; instead antihypertensive drugs have the above effect [55]. In our study, 8-week IMT showed similar differences between the groups in terms of pulmonary function. However, in the pretest-posttest intragroup comparison, FVC was different within the group in the LLG and HLG, and the PEF % value was different within all groups. Increasing respiratory muscle strength in HT patients may have improved respiratory functions as a result of a decreased diaphragmatic fatigue with respiratory metaboreflex activation. In addition, expiratory muscle strength is related to PEF values and the increase in PEF values might result from the improvement of respiratory muscle strength [56–58]. The metaboreflex effect of IMT may have improved respiratory functions in HT patients by affecting oxygenation. In order to prove the effectiveness of IMT on pulmonary function, more long-term training is needed.

### 4.6. Fatigue

The relationship between functional parameters such as lung mechanics, chest kinematics, metabolism, peripheral and respiratory muscle function, and exercise tolerance level remains a controversial issue. An increase in energy output required by an external stimulation results in fatigue, which is described as a disturbance of internal balance. Along with fatigue, physical performance declines as a result of a rise in the actual or perceived difficulty of a task or activity, as well as the muscles' inability to maintain the desired level of strength during exercises. Physical activity that results in the buildup of specific metabolites in muscle fibers or insufficient motor control in the motor cortex would be the stimulus for weariness [59]. Although it has been previously shown in other diseases that inspiratory muscle training can improve exercise tolerance in hypertensive patients, the degree to which each of the parameters mentioned above contributes to this change remains unclear [36, 60]. In our study, fatigue perception decreased statistically only within LLG. IMT led to an increase in both peripheral and respiratory muscle strength and increased exercise capacity in the groups, which may have led to a decrease in the perceived fatigue level. It is recommended to further investigate the relationship between fatigue level and respiratory exercises in hypertensive patients.

### 4.7. Physical Activity

Physical activity is widely recommended as an important lifestyle change that can help prevent HT. Epidemiologic evidence has shown a consistent, temporal, and dose-dependent association between physical activity and the development of HT. Experimental evidence has further confirmed the positive effects of exercise on lowering blood pressure have been well characterized in recent years [61]. In our study, more than half of the HT patients were inactive at baseline. At the end of 8 weeks, the physical activity level increased in the LLG and HLG. The effect of training was also observed in the changes in physical activity levels. Therefore, since high-intensity training seems to increase physical activity more, it is recommended to add this workload to exercise programs for patients with HT.

### 4.8. Blood Pressure, Dyspnea, and Sleep Quality

According to a review of the treatment efficacy of device-assisted breathing exercises in HT, based on studies of acceptable methodological quality, there is no clear evidence to support a short-term beneficial effect of device-guided breathing exercises on blood pressure [62]. However, a recent meta-analysis showed a reduction in systolic and diastolic blood pressure for respiratory muscle training when the load was applied (−15.72 (−18.63; −12.81) and −7.08 (−9.03; −5.13) mmHg, respectively). Respiratory muscle training without workload decreased the SBP, but not DBP (−5.08 (−7.49; −2.66) and −1.04 (−2.55; +0.46) mmHg, respectively) [33]. According to the results of our study, 8 weeks of breathing exercises training, including whether unloaded, low-load intensity, or high-load intensity training, decreased resting blood pressure in HT patients. SBP was lowered more by low-load intensity training, whereas DBP was lowered more by high-intensity training. However, no difference was found between the trainings. Therefore, as stated in the literature, breathing exercises are effective in lowering blood pressure with or without load. Respiratory and cardiovascular modulation work together significantly in regulating blood pressure, evidenced by their interdependent alterations in control mechanisms. This correlation is likely influenced by the sensitivity of baroreceptors and chemoreceptors, impacting blood pressure regulation mechanisms [5]. The decrease in resting blood pressure in HT patients who performed different IMT protocols may be due to these mechanisms. It is recommended to add IMT to HT treatment programs.

Dyspnea is a common symptom affecting up to 25% of patients seen in the ambulatory setting. It can be caused by many different underlying conditions and is sometimes a manifestation of a life-threatening disease. Problems with the cardiovascular system, such as hypertension, are a pathogenesis of dyspnea [63]. Patients with pulmonary hypertension (PH) frequently experience the debilitating feeling of dyspnea, particularly exertional dyspnea. While ignoring the role of respiratory control, the etiology of dyspnea in these patients has been linked to cardiovascular factors and specific anomalies of the respiratory system during exercise [64]. Hossein Pour et al. [65] found that weekly home-based IMT was effective and safe in reducing dyspnea and fatigue and improving the New York Heart Association's functional classification. According to Sağlam et al. [66], IMT provides significant improvements in respiratory muscle strength and exercise capacity, resulting in reduced dyspnea during activities of daily living and less fatigue in patients with PAH (pulmonary arterial hypertension). Similar to the literature, current study's finding showed that low-load IMT and high-load IMT could decrease dyspnea perception in HT.

In patients with hypertension, systolic blood pressure should be reduced to <140 mmHg and diastolic pressure to <90 mmHg and blood pressure should be around 130–139/80–85 mmHg [67]. Recent data from SPRINT recommend lowering blood pressure below 120/85 mmHg, but it is also expressed in the guideline recommendations to keep in mind that changes may not occur in the near future [68]. Although it is uncontrollable and frequently goes unnoticed, breathing is a crucial part of maintaining cardiovascular homeostasis. Respiratory training allows for the practice and improvement of respiration, which is characterized by diaphragmatic motions. Slow breathing lowers blood pressure and increases baroreflex sensitivity, according to an acute controlled breathing program with 6 cycles per minute compared to spontaneous breathing. After adopting slower breathing patterns, blood pressure decreases primarily due to autonomic and reflex mechanisms. Reducing respiratory cycles causes the lungs to expand more. In order to prevent lung overinflation, this mechanical change stimulates the Hering–Breuer reflex and pulmonary stretch receptors. This acts as an input to the medulla, which is a crucial area for cardiopulmonary reflexes and where data produced by arterial baroreceptors is gathered and consolidated. Thus, as required by the reflex mechanism, a vagal-mediated response is triggered in the presence of an acute rise in blood pressure and/or lung expansion. This response causes systemic vasodilation with a reduction in cardiac chronotropic and inotropic activities as well as a reduction in vascular peripheral resistance, which ultimately lowers blood pressure [69]. IMT decreases resting heart rate and blood pressure with the metaboreflex effect. According to a meta-analysis and review examining the effects of IMT on the cardiovascular system, this training was found to be particularly effective on heart rate and diastolic blood pressure [30]. According to a recently published study, IMT applied at high intensity (MIP 75%) reduced SBP by 9 ± 6 mmHg and DBP by 4 ± 4 mmHg. IMT-associated reductions in SBP and DBP occurred at week 2 of training (−4 ± 8 mmHg and −3 ± 6 mmHg, respectively) and persisted throughout the following 6-week intervention. Consistent with the literature, both low-load IMT and high-load IMT lowered blood pressure for 8 weeks. It is recommended that respiratory muscle training and breathing exercises be added to treatment programs as an alternative therapy, especially in patients with HT risk factors or diagnosed with HT.

There are a limited number of studies evaluating the effectiveness of breathing exercises on sleep quality. According to a meta-analysis study reviewing the effects of IMT in individuals with sleep apnea syndrome, IMT improved sleep quality, MIP, and respiratory functions [70]. Similar to the literature, eight-week IMT improved sleep quality in all groups, while the effect of training was similar. In conclusion, breathing exercises may have improved sleep quality through relaxation effects.

Comparing the effects of different IMT loads in HT patients, especially showing the effects on physical and psychological symptoms, and being performed only in patients with a diagnosis of HT (no comorbidities) are the strengths of the study. To evaluate exercise capacity with CPET, not examining the changes in cardiac functions in patients, and not showing the cost-effectiveness of IMT are the limitations of the study.

## 5. Conclusion

To the best of our knowledge, this is the first study that compared the different loads of IMT in HT patients. High-load IMT was particularly effective in increasing physical activity level, peripheral muscle strength, exercise capacity, and improved HrQoL. Low-load IMT was effective in reducing dyspnea and improving respiratory function. Device-guided breathing exercises when performed with load (%30 MIP and %50 MIP) or without workload (%10 MIP) decreased blood pressure, improved sleep quality, and strengthened respiratory muscles. It may be useful to take into account the effects of these different workloads when designing exercise programs for HT patients. Also, the efficacy of inspiratory muscle training should be investigated with different training protocols such as endurance IMT or functional IMT in HT patients.

## Figures and Tables

**Table 1:** Demographic, physical, and physiologic characteristics of HT patients.

Variables	CG (*n*: 15)	LLG (*n*: 15)	HLG (*n*: 15)	P value
	Mean ± SD/median (IQR)/*n* (%)	Mean ± SD/median (IQR)/*n* (%)	Mean ± SD/median (IQR)/*n* (%)	
Age (years)	59.13 ± 11.22	56.20 ± 6.39	59.86 ± 7.98	0.486
BMI (kg/m^2^)	26.69 ± 4.42	29.80 ± 3.11	28.75 ± 4.54	0.753
Gender (female/male)	4 (26.7)/11 (73.3)	1 (6.7)/14 (93.3)	2 (13.3)/13 (86.7)	0.463
*Level of education*				
Illiterate	1 (6.7)	1 (6.7)	1 (6.7)	0.911
Literate	2 (13.2)	0 (0)	5 (33.3)	
Primary education or less	4 (26.7)	5 (33.3)	3 (20)	
Secondary education	5 (33.3)	7 (46.7)	1 (6.7)	
High school education	1 (6.7)	1 (6.7)	3 (20)	
University	2 (13.3)	1 (6.7)	2 (13.3)	
*Marital status*				
Married/single/widowed	13 (86.7)/1 (6.7)/1 (6.7)	14 (93.3)/1 (6.7)/0 (0)	15 (100)/0 (0)/0 (0)	0.762
Occupation				
Retired/housewife/civil servant/worker/farmer/self-employed/driver	6 (40)/3 (20)/2 (13.3)/0 (0)/0 (0)/3 (20)/1 (6.7)	2 (13.3)/1 (6.7)/2 (13.3)/1 (6.7)/2 (13.3)/6 (40)/1 (6.7)	7 (46.7)/2 (13.3)/1 (6.7)/2 (13.3)/0 (0)/0 (0)/3 (20)	0.426
*Family medical history*				
HT	5 (35.7)	0 (0)	3 (23.1)	0.070
DM	3 (21.4)	4 (30.8)	5 (38.5)	0.630
CAD	6 (42.9)	7 (46.7)	2 (15.4)	0.189
*Physical activity status*				
Yes	7 (46.7)	5 (33.3)	5 (33.3)	0.795
No	8 (53.3)	10 (66.7)	10 (66.7)	
Smoking				
Current	3 (20)	4 (26.7)	4 (26.7)	0.832
Ex	5 (33.3)	7 (46.7)	7 (46.7)	
Nonsmoker	7 (46.7)	4 (26.7)	4 (26.7)	
*Alcohol consumption*				
Current	4 (26.7)	2 (13.3)	2 (26.7)	0.614
Ex	0 (0)	2 (13.3)	1 (6.7)	
No	11 (73.3)	11 (73.3)	12 (80)	
mMRC (0–4)	1 (1-1)	1 (1–1.5)	1 (1-1)	0.391
Resting MBS (0–10)	0 (0-1)	0 (0–0.5)	0 (0-1)	0.841
Activity MBS (0–10)	4 (3–5)	3 (1.25–6.5)	3 (2–4)	0.105
Exertional dyspnea	13 (86.7)	11(73.3)	11 (73.3)	0.734

CAD: coronary artery disease, DM: diabetes mellitus, HT: hypertension, mMRC: Modified Medical Research Council, MBS: Modified Borg Scale, and Kruskal–Wallis test/ANOVA/chi-square test, *p* < 0.05.

**Table 2:** The results of the effect of IMT on respiratory parameters in individuals with HT.

	CG (*n*: 15)			LLG (*n*: 15)			HLG (*n*: 15)			Treatment effect *p* value
	Pre mean ± SD	Post mean ± SD	Within group *p* value	Pre mean ± SD	Post mean ± SD	Within group *p* value	Pre mean ± SD	Post mean ± SD	Within group *p* value	
MIP (cmH_2_O)	74.46 ± 29.51	87.16 ± 35.19	**<0.029 ** ^*∗*^	85.46 ± 23.90	110.16 ± 28.80	**<0.001 ** ^*∗*^	88.53 ± 25.28	118.82 ± 30.45	**<0.01 ** ^*∗*^	0.054
MEP (cmH_2_O)	74.20 ± 22.27	84.26 ± 29.35	0.115	88.53 ± 24.09	109.97 ± 31.94	**0.001 ** ^*∗*^	93.80 ± 26.96	112.52 ± 41.23	**0.006 ** ^*∗*^	0.465
FEV_1_ (lt)	2.64 ± 0.62	2.55 ± 0.77	0.304	2.88 ± 0.83	3.01 ± 0.67	0.116	3.05 ± 0.83	3.09 ± 0.95	0.546	0.785
FEV_1_ (%)	81.50 ± 13	85.20 ± 16.62	0.338	88.86 ± 23.40	92.56 ± 15.68	0.092	91.20 ± 18.56	91.98 ± 18.98	0.485	0.764
FEV_1_/FVC (%)	84.92 ± 10.50	87.70 ± 12.96	0.159	86.71 ± 8.71	84.80 ± 6.58	0.459	82.70 ± 84.69	84.69 ± 9.24	0.467	0.296
FVC (lt)	2.86 ± 0.70	2.95 ± 0.78	0.867	3.42 ± 0.99	3.91 ± 1.24	**0.045 ** ^*∗*^	3.60 ± 1.27	4.26 ± 1.46	**0.006 ** ^*∗*^	0.113
FVC (%)	80.21 ± 14.10	83.80 ± 15.18	0.328	87.40 ± 23.11	89.82 ± 15.45	0.253	91.06 ± 21.62	90.69 ± 22.97	0.807	0.283
PEF (lt)	4.21 ± 1.62	5.43 ± 2.38	**0.011 ** ^*∗*^	5.24 ± 1.74	6.33 ± 2.26	**0.007 ** ^*∗*^	5.32 ± 2.05	6.01 ± 1.93	0.071	0.734
PEF (%)	57.78 ± 15.09	74.46 ± 22.52	**0.001 ** ^*∗*^	65.06 ± 20.90	77.23 ± 24.84	**0.005 ** ^*∗*^	68.73 ± 19.59	80.65 ± 17.82	**0.004 ** ^*∗*^	0.873
FEF_25–75_ (lt)	2.87 ± 1.24	3.01 ± 1.33	0.623	3.22 ± 1.09	3.52 ± 1.08	0.134	3.85 ± 1.52	4.03 ± 1.62	0.219	0.776
FEF_25–75_ (%)	75.71 ± 24.21	86.42 ± 27.73	0.040	86.53 ± 29.50	93.39 ± 29.50	0.110	92.86 ± 25.24	96.85 ± 28.41	0.269	0.762

FEV_1_: forced expiratory volume in 1 s; FVC: forced vital capacity; PEF: peak expiratory flow; FEF%_25–75_ (%): forced expiratory flow at 25–75% of the pulmonary volume; MIP: maximal inspiratory pressure; MEP: maximal expiratory pressure; ^*∗*^*p* < 0.05. Paired *t*-test. ANCOVA test.

**Table 3:** Comparisons of peripheral muscle strength, exercise capacity, HrQoL, depression, anxiety, sleep quality, physical activity status, dyspnea, and blood pressure within and between the groups.

	CG (*n*: 15)			LLG (*n*: 15)			HLG (*n*: 15)			Treatment effect p value
	Pre mean ± SD	Post mean ± SD	Within group p value	Pre mean ± SD	Post mean ± SD	Within;group p value	Pre mean ± SD	Post mean ± SD	Within group p value	
QF strength R (N)	167.26 ± 55.55	182.08 ± 50.16	0.299	180.33 ± 47.33	207.25 ± 46.07	**0.022**	182.80 ± 53.54	206.72 ± 76.08	**0.035**	0.606
QF strength L (N)	159.68 ± 62.51	168.86 ± 45.21	0.421	167.26 ± 48.20	201.81 ± 55.72	**0.001**	173.5 ± 47.97	195.50 ± 72.85	**0.026**	0.181
HG strength R (kgf)	62 ± 23.39	64.47 ± 22.53	0.531	66.66 ± 22.49	76.85 ± 19.31	**0.001**	78.80 ± 23.03	90.56 ± 28.16	<**0.001**	**0.039** ^*∗*^
HG strength L (kgf)	57.26 ± 16.70	60.65 ± 16.56	0.409	68.60 ± 21.10	71.82 ± 19.05	0.138	76.40 ± 23.58	81.25 ± 22.94	**0.011**	0.462
6MWT (m)	473.68 ± 67.64	496.13 ± 82.15	0.116	470.00 ± 102.101	537.55 ± 48.53	**<0.001**	490.92 ± 52.60	551.66 ± 52.04	**<0.001**	**0.020** ^*∗*^
SF-36 scale PF	65.66 ± 25.62	67.30 ± 25.27	0.500	71.33 ± 26.08	85.10 ± 15.84	**0.001**	86.33 ± 8.12	92.45 ± 7.67	**0.003**	**0.010** ^*∗*^
SF-36 scale PRF	61.66 ± 44.18	58.31 ± 44.22	0.481	64.66 ± 43.03	81.55 ± 38.58	0.068	76.66 ± 37.16	87.45 ± 35.63	0.088	0.132
SF-36 scale BP	54 ± 25.40	63.83 ± 20.02	0.943	80.50 ± 22.70	83.53 ± 24.92	**0.049** ^*∗*^	67.33 ± 20.81	83.46 ± 24	**0.008**	0.175
SF-36 scale SF	71.66 ± 27.33	70.77 ± 32.17	0.698	74.1 ± 34.54	76.57 ± 21.03	0.687	75.83 ± 23.36	88.78 ± 22.73	**0.031** ^*∗*^	0.177
SF-36 scale vitality	51.66 ± 20.23	56.42 ± 25.58	0.324	50.66 ± 24.26	61.71 ± 24.68	**0.033** ^*∗*^	50.66 ± 19.80	56.76 ± 23.20	0.232	0.661
SF-36 scale ERF	51.18 ± 46.94	59.28 ± 48.03	0.448	64.44 ± 46.23	73.92 ± 43.44	0.128	44.44 ± 49.86	74.68 ± 39.83	**0.008** ^*∗*^	0.355
SF-36 scale mental health	61.86 ± 15.91	62.94 ± 20.14	0.649	55.73 ± 19.15	70.68 ± 19.20	**0.002** ^*∗*^	54.66 ± 21.25	57.79 ± 23.75	0.606	0.083
SF-36 scale general health	54 ± 14.41	60.44 ± 20.24	0.206	60.66 ± 18.97	58.94 ± 18.40	0.925	52.33 ± 18.59	58.24 ± 20.45	0.323	0.690
HADS total score	8.46 ± 5.90	9.20 ± 6.98	0.402	6 ± 2.75	4.96 ± 4.25	0.144	9.64 ± 6.38	7.48 ± 4.81	0.104	0.143
PSQI total score	4.73 ± 1.70	4.69 ± 2.79	0.933	4.60 ± 1.68	4.33 ± 1.84	0.494	4.40 ± 1.99	4.12 ± 2.13	0.385	0.879
IPAQ total (MET-min/week)	964.53 ± 822.89	828.90 ± 781.72	0.503	1255.90 ± 1470.91	1321.17 ± 1423.35	0.753	1164.13 ± 1010.86	1893.03 ± 1766.95	**<0.001** ^*∗*^	**0.013** ^*∗*^
FSS total score	38.06 ± 17.81	31.68 ± 14.72	0.151	32.53 ± 13.45	24.46 ± 10.68	0.004^*∗*^	31.00 ± 13.87	30.29 ± 14.10	0.460	0.264
Dyspnea (rest. 0–10)	0.38 ± 0.96	0.46 ± 1.19	0.575	0.92 ± 0.20	0.19 ± 0.55	0**.012**^*∗*^	0.53 ± 1.30	0.07 ± 0.27	**0.009** ^*∗*^	0.244
SBP (rest. mmHg)	123.07 ± 13.15	122.21 ± 18.79	0.182	136.64 ± 12.25	130.83 ± 18.35	0.962	130.00 ± 19.14	132.05 ± 14.20	0.682	0.438
DBP (rest. mmHg)	74.23 ± 9.86	74.56 ± 10.27	0.352	84.85 ± 7.39	78.18 ± 12.95	0.151	85.83 ± 14.50	80.20 ± 16.11	0.370	0.926

QF, quadriceps femoris; HG, hand grip; 6MWT, 6-minute walk test; N, Newton; SF-36, short form 36; PF, physical functioning; PRF, physical role functioning; BP, bodily pain; SF, social functioning; ERF, emotional role functioning; HADS, Hospital Anxiety and Depression Scale; PSQI, Pittsburgh Sleep Quality Index; IPAQ, International Physical Activity Questionnaire; FSS, Fatigue Severity Scale; SBP, systolic blood pressure; DBP, diastolic blood pressure; ANCOVA test; ^*∗*^*p* < 0.05.

## Data Availability

The data sets used and/or analyzed during the study are available from the corresponding author (İH) upon reasonable request.
